# Physiologic and anatomic determinants of hyoid motion during drug-induced sleep endoscopy

**DOI:** 10.1007/s11325-024-03101-5

**Published:** 2024-07-11

**Authors:** Manan H. Parekh, Eric Thuler, Vasiliki Triantafillou, Everett Seay, Chandra Sehgal, Susan Schultz, Brendan T. Keenan, Alan R. Schwartz, Raj C. Dedhia

**Affiliations:** 1 Department of Otorhinolaryngology – Head & Neck Surgery, Perelman School of Medicine, University of Pennsylvania, 3400 Spruce Street, Philadelphia, PA 19104 USA; 2 Division of Sleep Medicine/Department of Medicine, University of Pennsylvania, Philadelphia, USA; 3 Department of Radiology, University of Pennsylvania, Philadelphia, USA

**Keywords:** Obstructive sleep apnea, Hyoid, Drug-induced sleep endoscopy

## Abstract

**Purpose:**

To examine factors accounting for differences in hyoid motion during obstructive breathing events amongst obstructive sleep apnea (OSA) patients.

**Methods:**

This was a prospective cohort study from June 2022 to October 2022. Patients with OSA undergoing evaluation for PAP alternative therapies with drug-induced sleep endoscopy with positive airway pressure titration (DISE-PAP). All patients underwent DISE-PAP and concurrent hyoid-focused ultrasound. DISE-PAP enabled measurement of airway physiology (flow, respiratory effort) and airway collapsibility (pharyngeal opening pressure, PhOP). Hyoid-ultrasound enabled hyoid bone movement during obstructive breathing. Respiratory effort was measured using a retro-epiglottic pressure-sensitive catheter. Hyoid position was measured using a standardized, awake, CT protocol. Regression analyses adjusted for age, race, sex, and BMI were performed to associate indices of respiratory effort and CT data with hyoid motion.

**Results:**

On average, the 26 patients in this cohort were older (63.9 ± 10.5 years), male (69%), overweight (29.6 ± 3.99 kg/m^2^), and with moderate-to-severe OSA (26.8 ± 10.4 events/hour). Greater respiratory effort was associated with increased hyoid motion (β [95% CI] = 0.034 [0.016,0.052], standardized β = 0.261,p = 0.0003). Higher hyoid position was associated with greater hyoid displacement (β [95% CI] = -0.20 [-0.38,-0.01], Standardized β = -0.57, *p* = 0.036).

**Conclusion:**

Our data demonstrate that greater respiratory effort, higher hyoid position, and higher airway collapsibility, but not airflow, are associated with greater hyoid motion during obstructive breathing in DISE. These findings suggest that downward hyoid movement represents a compensatory response to upper airway obstruction. Further studies should investigate the vectors of hyoid motion to better understand its role in sleep-related airway collapse.

**Supplementary Information:**

The online version contains supplementary material available at 10.1007/s11325-024-03101-5.

## Introduction

Obstructive sleep apnea (OSA) is a sleep-breathing disorder characterized by inspiratory flow limitation and is predisposed by both neuromuscular and anatomic vulnerabilities [1, 2]. Neuromuscular alterations can impact ventilatory control stability and arousal threshold, which have been implicated in OSA pathogenesis [2]. From an anatomical standpoint, craniofacial maldevelopment can lead to OSA through increased airway collapsibility [3, 4]. While the maxillo-mandibular boundaries affect the antero-posterior (AP) airway dimension, the hyoid position determines the cranio-caudal aspect. Cephalometric data suggest lower hyoid position correlates with increased airway collapsibility and OSA severity [5–7]. Additionally, the hyoid has been a surgical target for OSA clinicians, as studies have demonstrated that anterior hyoid positioning may improve airway patency [8, 9]. Overall, hyoid surgery for OSA has had mixed outcomes, highlighting a need for more comprehensive understanding of the hyoid in OSA pathophysiology [10, 11].

In addition to unfavorable static anatomic constraints, the dynamic behaviors of the upper airway structures have been implicated in OSA pathophysiology [9]. Several studies have suggested dynamic caudal movement of the hyo-laryngeal complex to be associated with airway patency [12, 13]. Tong et al. demonstrated tracheal displacement correlated with respiratory effort [14], and Pepin et al. showed mandibular motion correlated with increased respiratory effort during flow-limited breathing [15]. Given the established associations between static hyoid position and OSA, and its muscular and ligamentous attachments to the trachea and mandible, understanding hyoid motion in response to respiratory effort can inform its role in both OSA pathogenesis and treatment.

OSA patients presenting for surgical evaluation often undergo drug-induced sleep endoscopy (DISE) for evaluation of sites and severity of upper airway collapse [16, 17]. DISE with positive airway pressure titration (DISE-PAP) presents a controlled environment in which upper airway physiology and anatomy can be assessed, in both flow-limited and non-flow limited states. [18, 19] Our group recently leveraged DISE-PAP with concurrent hyoid-focused ultrasound to study hyoid dynamics. In a cohort of 20 OSA patients, we identified key features of dynamic hyoid displacement [20]. First, caudal hyoid displacement increased with obstructive breathing compared to non-obstructive breathing. Second, caudal hyoid displacement magnitude was associated with OSA severity (apnea–hypopnea index [AHI]). Finally, hyoid displacement magnitude during obstructive breathing varied amongst patients, prompting the exploration of physiologic and anatomic factors on hyoid mobility.

We leveraged this DISE-PAP protocol with submental ultrasound to investigate the variability in dynamic hyoid motion. Our primary aim was to evaluate the association between respiratory effort and dynamic hyoid motion in OSA patients during DISE. Specifically, we hypothesized that greater respiratory effort associates with greater hyoid displacement amongst patients. Our secondary aim was to evaluate the association between static hyoid position and hyoid motion during obstructive breathing. We hypothesized that more inferior hyoid position (posterior nasal spine – hyoid [PNS-H]) would correlate with increased hyoid mobility. Finally, we aimed to explore the relationship between dynamic hyoid motion and airway collapsibility, extra-hyoid craniofacial dimensions (via computerized tomography [CT]), and nasal airflow.

## Methods

### Participants

We performed a cross-sectional analysis from a prospective single-point cohort of patients previously diagnosed with OSA via polysomnography or home sleep apnea testing [respiratory polygraph) seeking PAP alternatives due to unwillingness to use CPAP or intolerance to therapy. All patients underwent DISE-PAP and submental ultrasound and standardized awake CT scans at the Hospital of the University of Pennsylvania between June 2022 and October 2022. The study was approved by Institutional Review Board at the University of Pennsylvania (IRB*#:* 849542). Inclusion criteria were age greater than or equal to 18 years and apnea–hypopnea index (AHI) > 5 events/hour. Patients were excluded due to either inadequate quality or quantity of recordings or signals captured.

### Clinical data

Demographic and anthropometric information including sex, age, weight, height*,* BMI, and race were obtained from the electronic medical record.

### CT sleep protocol

CT scans were obtained using a standardized protocol focused on the upper airway as previously described [4]. In brief, CT scans were performed at kV 120, with slice thickness < 1 mm, pitch of 1.0, FOV of 25 cm, and no gantry tilt. Thin reconstructions were obtained in axial, sagittal, and coronal planes. Patients were instructed to lie down on their back on a flat table with their head and neck in neutral position. Patients were asked to smile with teeth in occlusion, exhale through their nose, then hold their breath during CT acquisition. Craniofacial CT measurements were performed following an established protocol by a physician (ET) blinded to clinical data [4].

### DISE-PAP procedure

The DISE-PAP protocol was performed on an integrated recording platform designed to acquire clinically relevant anatomic and physiologic characteristics of each patient’s upper airway. A detailed description of the DISE-PAP setup has been previously published [18]. In short, the nasolaryngoscope was passed through a custom-fitted mask into the nasal cavity. Masks were either nasal or oro-nasal, depending on resource availability. All CPAP (S9 VPAP, ResMed Inc., San Diego CA) was applied using a nasal interface (Pulmodyne, Indianapolis, IN) attached to a pneumotachometer. A pressure-sensitive catheter (Mikro-cath™, Millar, Houston, TX) was passed intra-nasally to the retro-epiglottic space to measure respiratory effort [21]. Propofol anesthesia was administered to achieve sedation using a probability ramp infusion system as previously described, with a target Bispectral Index (BIS) (Medtronic, Minneapolis, Minnesota) range of 50 – 70 [22]. Following completion of the baseline exam with observation of obstructive apneas, hypopneas, and/or flow limitation, nasal PAP was titrated starting at ~ 2 cm H_2_O and increased by 1 cm H_2_O during respiratory arousals until flow-limitation was abolished for > 50% of breaths at a given nasal pressure level. This is known as the pharyngeal opening pressure (PhOP), a measure of upper airway collapsibility [19, 23].

### Ultrasound acquisition and analysis protocol

Ultrasound recordings were obtained during DISE with a Zonare S3 scanner (Mindray North America, Mahwah, NJ, USA) using a curved array C6-2 probe positioned submentally in the mid-sagittal plane. The scans were performed after a stable sedation plane was achieved with observation of obstructive respiratory events. Care was taken to ensure probe pressure on the submental space was minimal to avoid influencing upper airway collapse or mouth position. Hyoid displacement during inspiration was measured using Imagelab (L3HarrisGeospatial, Colorado, US). Absolute magnitude of hyoid displacement per breath was measured along its linear vector of motion. Each measurement was done in triplicate, then mean hyoid displacement amplitude was calculated by averaging hyoid motion values over the number of breaths recorded. A detailed description of our acquisition and analysis protocol for hyoid displacement is included in the attached supplement (S1).

### Measuring respiratory effort and airflow

DISE pressure-flow tracings were analyzed using LabChart 8 (ADInstruments, Colorado Springs, CO). Our DISE-PAP recordings were imported, and the breaths captured by ultrasound were located. Respiratory effort was defined as the pressure differential between inspiratory start and nadir of negative pressure during inspiration (cm H_2_O). Nadir was defined as the greatest negative value of pressure noted during the inspiratory phase. The pressure differential was calculated for each breath captured by ultrasonography. Individual breaths were excluded due to retro-epiglottic catheter interference resulting from catheter dislodgement during DISE or excess mucous. Flow-limited breaths were categorized based on a visual evaluation of airflow and effort: obstructive apneas or hypopneas.

### Statistical analyses

Continuous or categorical variables are summarized using means and standard deviations (SDs) or frequencies and percentages, respectively. Linear mixed-effects modeling analysis accounting for repeated breaths per person was used to explore the relationship between per-breath respiratory effort and per-breath mean hyoid displacement. A repeated measures correlation coefficient was calculated as a complementary descriptor of the association. Linear regression analysis was used to associate cephalometric data of hyoid position, pharyngeal opening pressure (PhOP), and patient-level average hyoid motion (averaged across all breaths per patient). Regression analyses were conducted controlling for age, sex, body mass index (BMI), and height; complementary unadjusted associations were also evaluated. Results are reported as β-coefficients and associated 95% confidence intervals (CIs), representing the expected change in outcome for a one unit increase in the predictor. In addition, standardized β-coefficients equal to the estimated SD change in outcome for 1 SD increase in predictor are presented; estimates of 0.2, 0.5, and 0.8 generally represent small, medium, and large effect sizes [24]. A p < 0.05 was used to determine statistical significance in primary analyses associating respiratory effort, hyoid position (PNS-H), and airway collapsibility with hyoid motion. For other association analyses, a Hochberg step-up procedure was used to determine statistical significance while maintaining overall type I error at 5% in the context of multiple comparisons; a p < 0.05 was considered nominally significant [25]. Analyses were performed using Stata/SE v14.3 (StataCorp, College Station, TX) and SAS Version 9.4 (SAS Institute, Cary, NC).

## Results

### Participant characteristics

39 patients underwent DISE-PAP with concurrent hyoid-focused ultrasound. Of these patients, 26 met our inclusion criteria and 13 were excluded: 8 (20.5%) patients had poor ultrasound technique and probe motion, 3 (7.6%) had less than three breaths available per clip, 1 patient had technical limitations with ultrasound acquisition (2.5%), and 1 (2.5%) had excessive facial hair precluding ultrasound probe contact with the neck. Table 1 shows the baseline characteristics of our study sample. On average, patients were older (63.9 ± 10.5 years), male (69%), overweight (29.6 ± 4.0 kg/m^2^), had moderate-to-severe OSA (AHI 26.8 ± 10.4 events/hour, ODI 26.4 ± 11.8 events/hour), and had a mean PAP level during DISE of 5.2 ± 1.8 cm H_2_O.

**Table 1:** Sample characteristics (*n* = 26)

Variable	Estimate^†^
Age, years	63.9 ± 10.5
Male, *n* (%)	18 (69.2%)
Body Mass Index, kg/m^2^	29.6 ± 4.0
Height, m	1.74 ± 0.08
Apnea Hypopnea Index (AHI), events/hour	26.8 ± 10.4

^†^estimates presented as mean SD for continuous measures or *n* (%) for categorical data

A table depicting which patients were included for each sub analysis is shown in S2.

### Respiratory effort is associated with caudal dynamic hyoid displacement amplitude

**Fig. 1:**
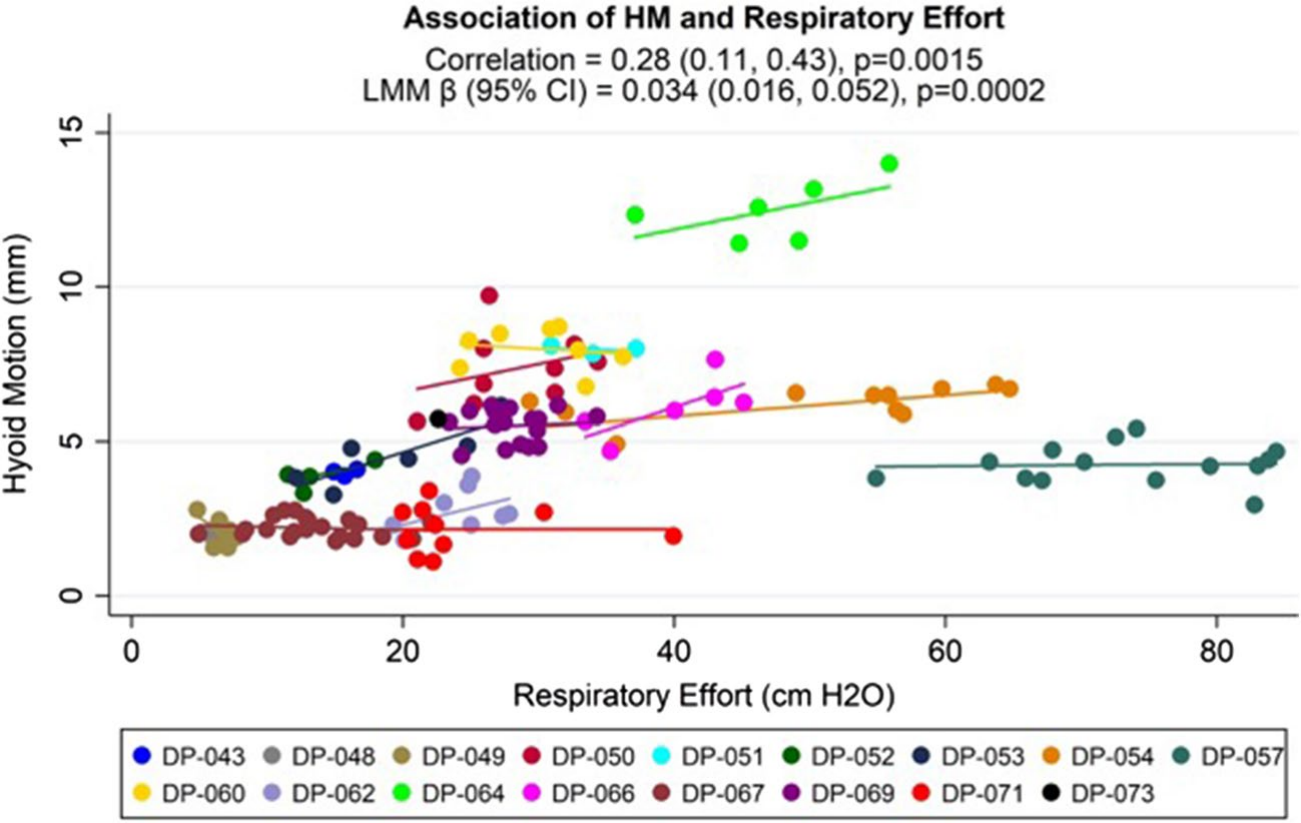
Association between Respiratory Effort and Hyoid Motion (HM). X axis shows negative pressure differential (cm H_2_O). Y axis shows per-breath hyoid motion (mm). Each point represents an individual breath measured within a patient

A total of 253 breaths were analyzed across 26 patients during obstructive breathing during DISE-PAP. After exclusions due to catheter dislodgement or mucous interference during ultrasound recording, 144 breaths (56.9% of all breaths) across 17 patients (65.4% of all patients) were included for respiratory effort analysis against hyoid motion amplitude. Individuals in whom respiratory effort could not be obtained (n = 9) had a 6 kg/m^2^ lower BMI on average (p = 0.001) than those with observed data (n = 17), but had similar age, sex, height, AHI, and hyoid displacement (S3). The mean respiratory effort pressure differential was 29.6 ± 19.6 cm H_2_O, and the mean amplitude of dynamic caudal hyoid displacement for these breaths was 4.6 ± 2.6 mm. In repeated measures correlation analysis, there was a statistically significant association between amount of respiratory effort and amplitude of caudal hyoid motion across patients (r = 0.28, p = 0.0015). In covariate adjusted linear mixed model analysis, for every 1 cm H_2_O increase in respiratory effort there was a 0.034 mm increase in dynamic caudal hyoid displacement (95% CI = 0.016, 0.052; p = 0.0003), with a standardized effect size of 0.261 (95% CI: 0.120, 0.402) (see Fig. 1). Thus, our data demonstrate that increased effort is associated with more hyoid displacement during obstructive breathing.

### Inferior hyoid position is associated with lower mean caudal dynamic hyoid displacement amplitude

Twenty-three patients (88.5% of all patients) had CT data and measurements of posterior nasal spine to hyoid body (PNS-H) during DISE-PAP. Mean PNS-H was 74.6 ± 9.5 mm. Mean hyoid displacement for this subset was 5.7 ± 3.3 mm. Unadjusted analyses are presented in (S4). After adjusting for age, sex, BMI, and height, every 1 mm increase in PNS-H was associated with a 0.20 mm reduction (95% CI: 0.01, 0.38; p = 0.036) in dynamic hyoid motion, with a moderate standardized effect size of 0.57 (95% CI: 0.04, 1.11). Thus, increased PNS-H (e.g., lower hyoid position) was associated with less hyoid movement. Other measures relating to hyoid position and hyoid dynamics are shown in Table 2; consistent associations were seen with mandibular plane to hyoid distance and hyoid angle, although results were not statistically significant after Hochberg correction.

**Table 2:** Association between Hyoid Position and Hyoid Displacement (*n* = 23)

Hyoid Position Measure	β (95% CI)^†^	Std. β (95% CI)^‡^	*p*-value
PNS-H (mm)	-0.20 (-0.38, -0.01)	-0.57 (-1.11, -0.04)	0.036
MP-H (mm)	-0.30 (-0.55, -0.06)	-0.63 (-1.14, -0.12)	0.019
Hyoid angle (˚)	0.10 (0.01, 0.19)	0.58 (0.08, 1.08)	0.026
C3-Hyoid Distance (mm)	-0.05 (-0.48, 0.38)	-0.09 (-0.85, 0.66)	0.797
Mandible-Hyoid Distance (mm)	-0.03 (-0.33, 0.26)	-0.07 (-0.67, 0.54)	0.819

*PNS-H *Posterior Nasal Spine – Hyoid, *MP-H *Mandibular Plane – Hyoid

^†^Beta-coefficient from linear regression adjusted for age, sex, BMI and height, equal to the expected change in hyoid motion for 1 unit increase in hyoid position measure

^‡^standardized beta representing the expected SD change in hyoid motion for 1 SD increase in hyoid position measure, with values of 0.2, 0.5 and 0.8 representing small, medium and large effects

### Exploring pharyngeal opening pressure (PhOP), airflow, craniofacial measurements, and mean dynamic hyoid displacement amplitude

**Fig. 2:**
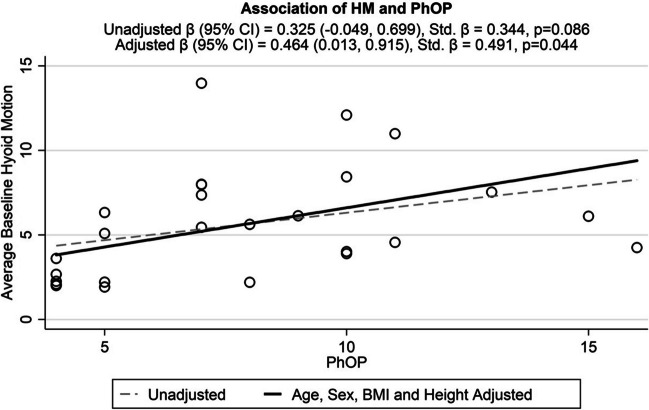
Association between Pharyngeal Opening Pressure (PhOP) and Hyoid Motion (HM). X axis shows PhOP (cm H_2_O). Y axis shows per-patient mean hyoid motion (mm)

In our adjusted models, we found increased airway collapsibility (PhOP) to associate with greater hyoid displacement amplitude (β [95% CI] = 0.46 [0.01, 0.92]; standardized β [95% CI] = 0.49 [0.01, 0.97]; p = 0.044) (Fig. 2). There was no association between categorized airflow (obstructive apneas or hypopneas) and hyoid displacement amplitude (S5). Individual breaths compared between nasal apnea and hypopnea are shown in S6. There was no association between extra-hyoid craniofacial measurements (S7) and hyoid displacement amplitude.

## Discussion

This is the first study investigating physiologic and anatomic contributors to dynamic hyoid motion in OSA patients during DISE. Our investigation informs of key factors associated with hyoid displacement during drug-induced sleep. First, increased respiratory effort during obstructive breathing was associated with increased caudal hyoid displacement magnitude. Second, higher hyoid position (decreased PNS-H) in the upper airway was associated with more dynamic caudal hyoid displacement. Further, increased upper airway collapsibility (PhOP) was associated with greater dynamic hyoid displacement. Finally, in binary categorization of obstructive events (apneas vs. hypopneas), there was no relationship between nasal airflow and hyoid displacement amplitude during obstructive breathing.

Several studies have investigated the relationship between respiratory effort and movement of upper airway structures. First, using awake dynamic MRI in 34 participants, Tong et al. investigated the relationship between dynamic caudal tracheal displacement and retro-epiglottic pressure nadir [14]. They found tracheal displacement amplitude to correlate with epiglottic pressure nadir (r = 0.47, p = 0.023). Our correlation of dynamic hyoid motion with respiratory effort (r = 0.28, p = 0.003) aligns with this existing literature. In adult male rabbits, also with a freely suspended hyoid bone, Amatoury et al. investigated hyoid displacement relative to manual caudal tracheal displacement [26]. For every 1 mm of caudal tracheal displacement, the hyoid moved 0.22 mm caudally. As the hyoid is also attached to various suprahyoid structures, the counterforce of suprahyoid attachments likely accounts for the smaller correlation with respiratory effort seen in the present study. Finally, Pepin et al. compared respiratory effort and mandibular motion across distinct respiratory events (e.g. normal breathing, obstructive events, central events), and found mandibular motion to tightly correlate with respiratory effort within a given event [15]. Our observation of a positive relationship between respiratory effort and hyoid motion during obstructed breathing on the population level is consistent with and extends these event-level observations. Notably, while we observed a significant relationship between effort and hyoid motion (r^2^ = 0.078), we identified other determinants influencing hyoid mobility during obstructive breathing including static anatomy, collapsibility, and airflow.

The position of the hyoid bone is influenced by tongue volume, obesity, and/or lengthening of hyoid ligaments over time [27, 28]. Furthermore, inferior hyoid position is associated with OSA severity and airway collapsibility [4–6]. On the other hand, the degree of hyoid movement during obstructive breathing may be influenced by the competing force vectors of its superior and inferior muscular attachments. We posited a static inferior hyoid bone position to be related to the lengthening of hyoid ligaments from recurrent, nightly increased respiratory effort, which would be reflected in a relationship between lower hyoid position and greater mobility [27]. Given our unexpected finding that higher static hyoid position on CT was associated with greater hyoid mobility, our data suggest recurrent hyoid motion does not lead to inferior hyoid position. When the hyoid is in a lower position, “stretching” of suprahyoid muscle fibers may instead limit the potential of downward hyoid motion. Additionally, low hyoid position may result in the vectors of suprahyoid muscular contraction to be in direct opposition with the caudal vector of tracheal motion, reducing the overall downward motion of the hyoid. Future studies should evaluate suprahyoid muscular activity and hyoid motion to better describe the relationship between hyoid position and motion.

We found increased airway collapsibility (PhOP) to be associated with greater caudal hyoid displacement during obstructive breathing. As greater collapsibility is associated with more severe OSA, this finding is consistent with our group’s previously shown relationship between AHI and hyoid motion amplitude. Studies have demonstrated caudal tracheal tension to reduce airway collapsibility, redistribute extraluminal pressures, and improve airway patency [12, 13, 29]. Thus, patients with greater caudal hyoid displacement during obstructive breathing may be compensating for a greater anatomic load (increased collapsibility).. Taken together, our data suggest that the magnitude of caudal hyoid motion is related to the severity of airway obstruction, both in terms of effort and collapsibility. Of note, our ultrasound methodology did not examine force vectors of hyoid motion. In their investigation of electrical stimulation of hyoid muscles in dogs, Van de Graaf et al. hypothesized the sum of suprahyoid and infrahyoid force vectors to result in a beneficial, antero-inferior, hyoid motion which reduces airway resistance [9]. A recent investigation by Kent et al. in eight OSA patients has supported this premise by showing inspiratory flow max to increase when adding ansa cervicalis electrical stimulation to hypoglossal nerve stimulation [30]. Dynamic sleep MRI, unlike ultrasound, may provide a testbed for this proposed mechanism as it enables visualization of both anterior–posterior and cranio-caudal vectors of hyoid motion during obstructive breathing.

This study has notable limitations. First, this was a small cohort of OSA patients seeking PAP-alternatives, limiting the generalizability of these findings. Similarly, we were unable to replicate previously established relationships between hyoid position, collapsibility, and OSA severity in our dataset. With regards to our ultrasound acquisition protocol, there were many initial exclusions due to protocol changes made to optimize acquisition and analysis. In addition, while minimal pressure was applied to minimize the influence of the ultrasound probe on airway physiology, this possibility cannot be ruled out. The mask type used during DISE also presents a limitation. Nine patients (34.6%) had oro-nasal masks while the rest had nasal masks. Although masks were selected based on resource availability, all CPAP during DISE was delivered via nasal port. Mask choice may have had an influence on mouth position which can alter measurements obtained during the study. Further, AP and cranio-caudal axes of hyoid motion could be analyzed with ultrasound. The change of hyoid position from static awake supine CT to propofol induced sleep was not assessed and may be influenced by other factors such as lung volume. Other limitations inherent to DISE are noteworthy, including sedation plane stability, comparisons to natural sleep, and influence of propofol on muscular activity. With regards to physiologic recordings, the retro-epiglottic pressure catheter limited the sample size for this analysis due to dislodgement or mucous interference. Finally, we did not assess suprahyoid muscular activity with electromyogram (EMG) during obstructive breathing.

This study bears several strengths as well. First, our previous work has shown ultrasound during DISE to be a reliable method for evaluating hyoid bone motion with high inter-rater reliability. Next, DISE-PAP offers a controlled setting, which enables standardized assessment of airway physiology. Our DISE-PAP protocol enables synchronous view of ultrasonography, physiology, and endoscopy, to better characterize airway dynamics. DISE-PAP also enables an objective measurement of airway collapsibility (PhOP). Our CT protocol is also a standardized method of assessing static anatomy of OSA patients.

**Fig. 3:**
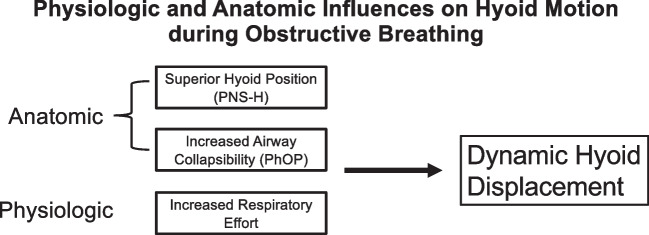
Factors which influence magnitude of dynamic hyoid motion during obstructive breathing. Anatomic influences were PNS-H distance and PhOP. The main physiologic influence was respiratory effort

Our investigation is the first to evaluate factors associated with dynamic hyoid motion during obstructive breathing in OSA patients during DISE. Increased respiratory effort, higher static hyoid position, greater airway collapsibility, but not airflow are key features which influence hyoid mobility (Fig. 3). These findings suggest that downward hyoid movement represents an active, neuromuscular response to upper airway obstruction. Future studies should assess anterior–posterior and cranio-caudal vectors of hyoid movement during obstruction to better characterize the role of hyoid dynamics in OSA patients.

## Supplementary Information

Below is the link to the electronic supplementary material.

Supplementary file1 (DOCX 1332 KB)

## Data Availability

The demographic and clinical data presented in Table 1, are available on request. The CT data are also available on request. The raw image files, however, are not as they contain patient-identifying information, protected under the Health Insurance Portability and Accountability Act (HIPAA). The data obtained for Figs. 1–2, and in the Supplemental Materials are available on request to the authors.
