# Supplementary material for: Physiologic and anatomic determinants of hyoid motion during drug-induced sleep endoscopy
Source: Sleep Breath. 2024 Jul 11;28(5):1997–2004. doi: 10.1007/s11325-024-03101-5 (PMC11450060; doi:10.1007/s11325-024-03101-5)
Supplement: Supplementary file 1 — Supplementary file1 (DOCX 1332 KB) [file 11325_2024_3101_MOESM1_ESM.docx]

Supplement: The Influence of Physiology and Anatomy on Hyoid Motion during Drug Induced Sleep Endoscopy

**S1**: Hyoid Displacement Quantification Methodology

***Acquisition***

Ultrasound recordings were obtained with a Zonare S3 scanner (now Mindray North America). The probe was a curved array C6-2. Once drug-induced sleep was achieved, and a stable sedation plane (BIS level between 50-70) was observed by the attending surgeon and anesthesiologist, the ultrasound probe was placed in position. The probe tip was carefully placed at the symphysis of the mandible, and minimal pressure was applied to avoid changing mouth position during DISE. After observation of obstructive hypopneas and apneas, the ultrasound recording was initiated. Ultrasound clips were between 15-60 seconds long, depending on other circumstances related to DISE (prolonged apnea requiring rescue arousal, and time constraints).

After the clip was obtained, a quality check was performed by an additional ultrasonographer to ensure good probe stability, contact between probe and skin, and hyoid visibility.

***Ultrasound Orientation***

The ultrasound probe tip was placed at the symphysis of the mandible. On ultrasound clips captured, the “Z” point marks the probe tip. Bone on ultrasound casts a hypoechoic shadow behind it, thus leading to a sharp contrast between the mandible-mandible shadow. Similarly, the hyoid has a contrast between the hyoid-hyoid shadow interface.

***Analysis***

The ultrasound clip was viewed in “Noxturnal US”. This software allows synchronized view of the endoscopic image, physiologic measure of flow, and ultrasonography (S1.1). The direction of hyoid displacement and timing of hyoid displacement relative to breath cycle was determined by corroborating with endoscopic recordings and physiologic data. This was confirmed by two researchers.


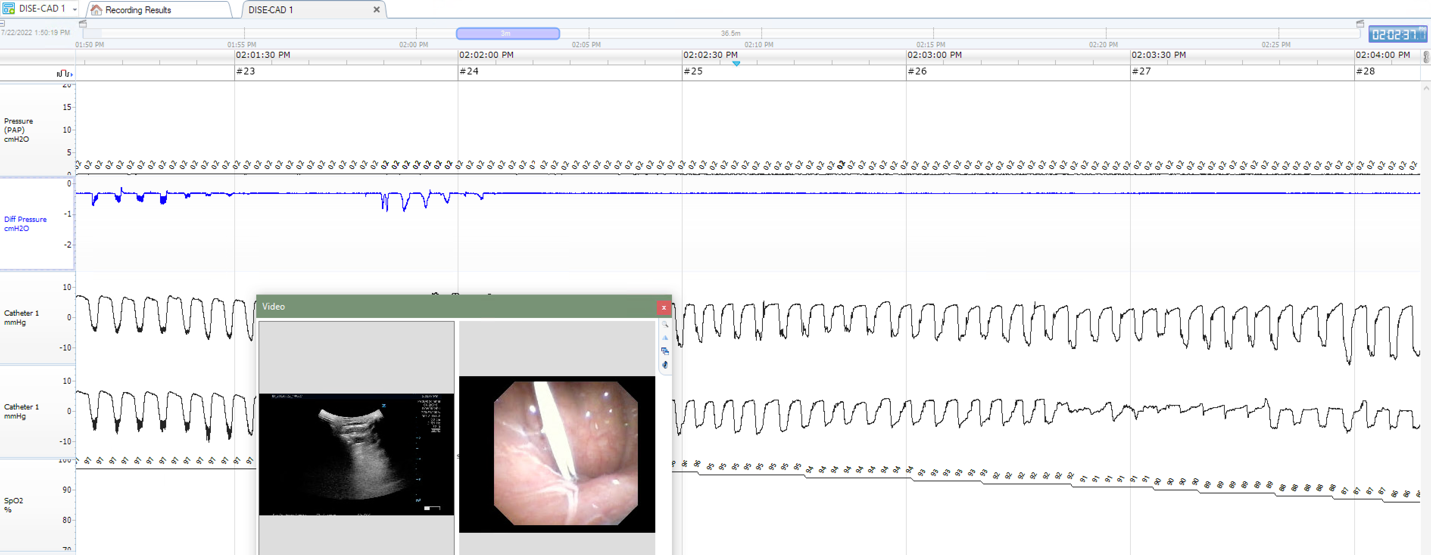


S1.1: Starting from the left, “Pressure (PAP) cm H2O” denotes the level of PAP applied to the patient. “diff pressure cm H2O” represents nasal flow. Beneath, “catheter 1 mmHg” represents the retro-epiglottic pressure catheter. The second “catheter 1 mmHg” represents the retropalatal pressure catheter. “SpO2 %” represents oxygen saturation. The two images are: ultrasound on the left, and endoscopy on the right.

Arousal breaths were identified as breaths occurring after hypopneas/apneas with reduction of effort and >20% increase in airflow. Central events were defined as a 10 second or longer cessation of breathing in the absence of respiratory effort. Arousal breaths and central events were noted and excluded from analysis.

Ultrasound clips were then uploaded to a proprietary software, Imagelab, developed using the programming language IDL (L3Harris Geospatial, Colorado, USA). First, the pixel size was calibrated using the cm scale on the image (S1.2).


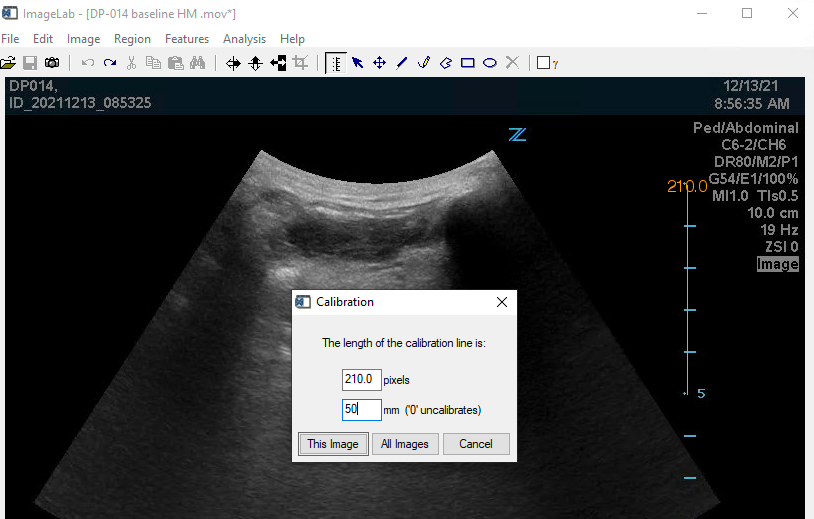


S1.2: In IDL, the calibration window allows the appropriate pixel:mm scale to be set.

Following calibration, the clip was played again in IDL to visualize the vector of hyoid as it moved. The gamma level of the ultrasound clips was adjusted to optimize contrast between hyoid and its shadow. By watching the ultrasound clip, initial position and position at end-inspiration (maximal hyoid displacement) were noted. Using the “polygon” tool, a vertical line was drawn starting from the midpoint of the hyoid bone surface extending inferiorly. One line was drawn at the hyoid’s baseline position. Another line was drawn in the same fashion at position of maximal displacement (S1.3).

Then, a third line was drawn connecting the starting points of the initial vertical marking lines. The third line was drawn and intentionally extended beyond the baseline and maximal displacement points of the hyoid to ensure motion was adequately captured.


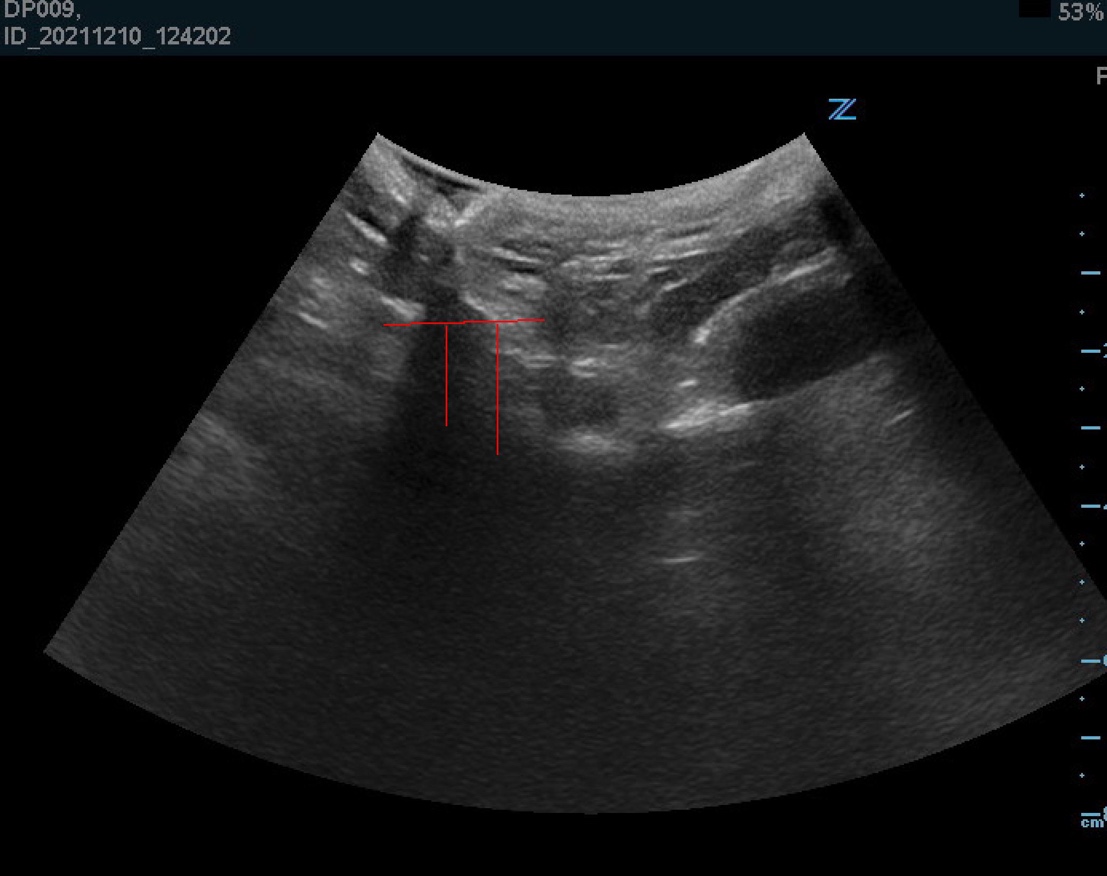


S1.3: Initial hyoid position and position after maximal displacement are marked. The connecting vector represents directionality of motion.

The lines were drawn such that they were contained within the same part of the identified hyoid bone. Hyoid bone identification was done by two raters.

The line was copied and pasted to all frames of the ultrasound clip. Then, the initial two vertical marking lines were deleted. Using the “virtual m-mode” function, displacement of the hyoid-hyoid shadow interface as it related to the line representing hyoid motion was analyzed in each frame of the ultrasound clip and compiled into a graph called a “motiongram” (S1.4)


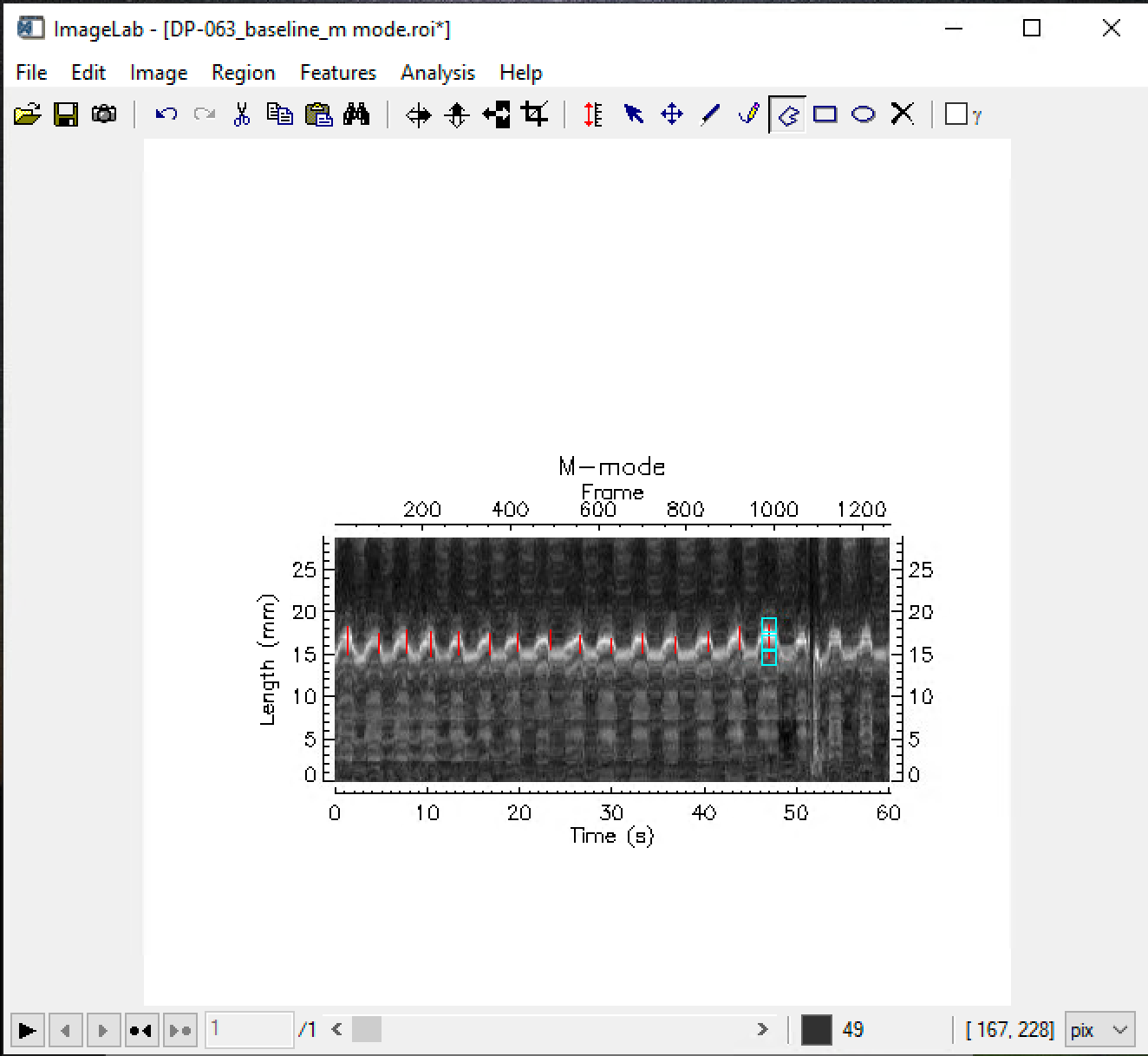


S1.4: A motiongram obtained during obstructive breathing is shown. The x axis represents time in seconds along the bottom, and frames along the top. The y axis represents distance in millimeters.

***Measuring the motiongram***

The motiongram “.bmp” file was opened using “imagelab”. Again, the calibration tool was used to set a pixel:mm ratio based on the y-axis of the motiongram. The hyoid was retroactively identified and measured along the initial connecting vector to obtain an understanding of where it would appear along the motiongram y axis. The absolute displacement of hyoid position was measured by capturing the tip of hyper-echogenicity at peak displacement to the tip of hyper-echogenicity at baseline position. Using the polygon tool, a vertical line was drawn from peak displacement to baseline position. Then, the measurements were batch extracted and pasted into an excel sheet. This was done in triplicate for both flow-limited and non-flow-limited recordings.

Patients with less than three breaths per ultrasound clip were excluded. Ultrasound clips that demonstrated significant lack of contact between probe and skin over important landmarks were also excluded. Additionally, breaths were excluded from analysis on motiongram when baseline position of the hyoid differed by +/- 4 mm from the breath immediately preceding or breath immediately after. Positional change greater than 4 mm were due to probe motion and therefore excluded. Finally, any central apneic events were excluded due to the study aim of evaluating obstructive sleep apnea instead of central sleep apnea.

**S2**: Patients Included per Analysis Condition

| **Analysis Arm** | **Included patients** | **Excluded patients** |
| --- | --- | --- |
| *Hyoid motion vs Respiratory Effort* | 17 | 9 |
| *Hyoid motion vs CT* | 23 | 3 |
| *Hyoid motion vs nasal flow* | 26 | 0 |
| *Hyoid motion vs PhOP* | 26 | 0 |

Table S2: Table depicting how many patients were included or excluded for each sub-analysis performed during this study.

**S3:** Included and Excluded Patients for Comparison of Respiratory Effort and Hyoid Motion

| **Variable** | **Excluded**  (n=9) | **Included**  (n=17) | **p** |
| --- | --- | --- | --- |
| *Age (years)* | 63.7 ± 14.6 | 64.1 ± 8.2 | 0.94 |
| *Male* | 66.7% | 70.59% | 0.84 |
| *BMI (kg/m^2^)* | 25.7 ± 3.6 | 31.7 ± 2.3 | 0.0008 |
| *Height (m)* | 1.74 ± 0.05 | 1.74 ± 0.09 | 0.91 |
| *AHI (events/hour)* | 23.4 ± 11.2 | 28.6 ± 9.8 | 0.51 |
| *Hyoid Motion (mm)* | 6.72 ± 4.01 | 5.07 ±2.72 | 0.29 |

Table S3: Characteristics of patients that were included and excluded in analysis of respiratory effort and hyoid motion.

**S4:** Unadjusted Analyses between Hyoid Position and Hyoid Motion

| **Hyoid Position Measure** | **β (95% CI)**^†^ | **Std. β (95% CI)**^‡^ | **p-value** |
| --- | --- | --- | --- |
| ***PNS-H (mm)*** | -0.09 (-0.25, 0.06) | -0.28 (-0.73, 0.17) | 0.21 |
|  |  |  |  |
| *MP-H (mm)* | -0.16 (-0.38, 0.05) | -0.34 (-0.78, 0.10) | 0.12 |
| *Hyoid angle (˚)* | 0.06 (-0.02, 0.14) | 0.34 (-0.10, 0.78) | 0.12 |
| *C3-Hyoid Distance (mm)* | 0.05 (-0.22, 0.31) | 0.08 (-0.38, 0.55) | 0.72 |
| *Mandible-Hyoid Distance (mm)* | -0.01 (-0.24, 0.22) | -0.03 (-0.49, 0.44) | 0.91 |

Table S4: Beta-coefficient from unadjusted linear regression equal to the expected change in hyoid motion for 1 unit increase in hyoid position measure; ^‡^standardized beta representing the expected SD change in hyoid motion for 1 SD increase in hyoid position measure, with values of 0.2, 0.5 and 0.8 representing small, medium and large effects. Abbreviations: PNS-H = Posterior Nasal Spine – Hyoid. MP-H = Mandibular Plane – Hyoid

**S5:** Hyoid motion based on nasal flow or no flow**
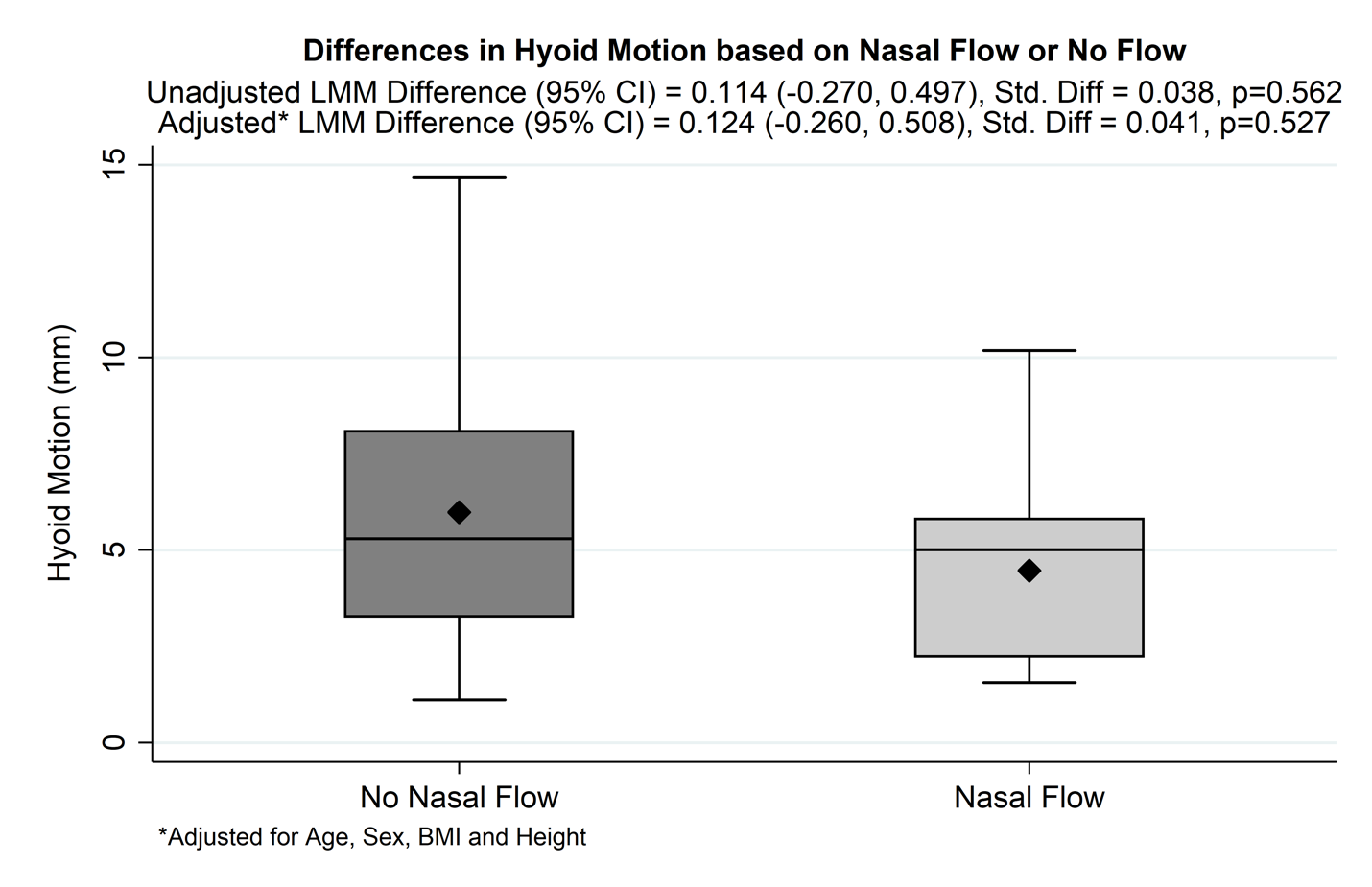
**

**Figure S5:** Association between Nasal Airflow and Hyoid Motion. X axis shows binary categorization of flow during obstructive breathing (presence/absence). Y axis shows a box plot of hyoid motion (mm). The diamond signifies mean hyoid motion in each condition.

**S6:** Hyoid Motion of Individual Breaths Measured during Nasal Apnea or Hypopnea

**
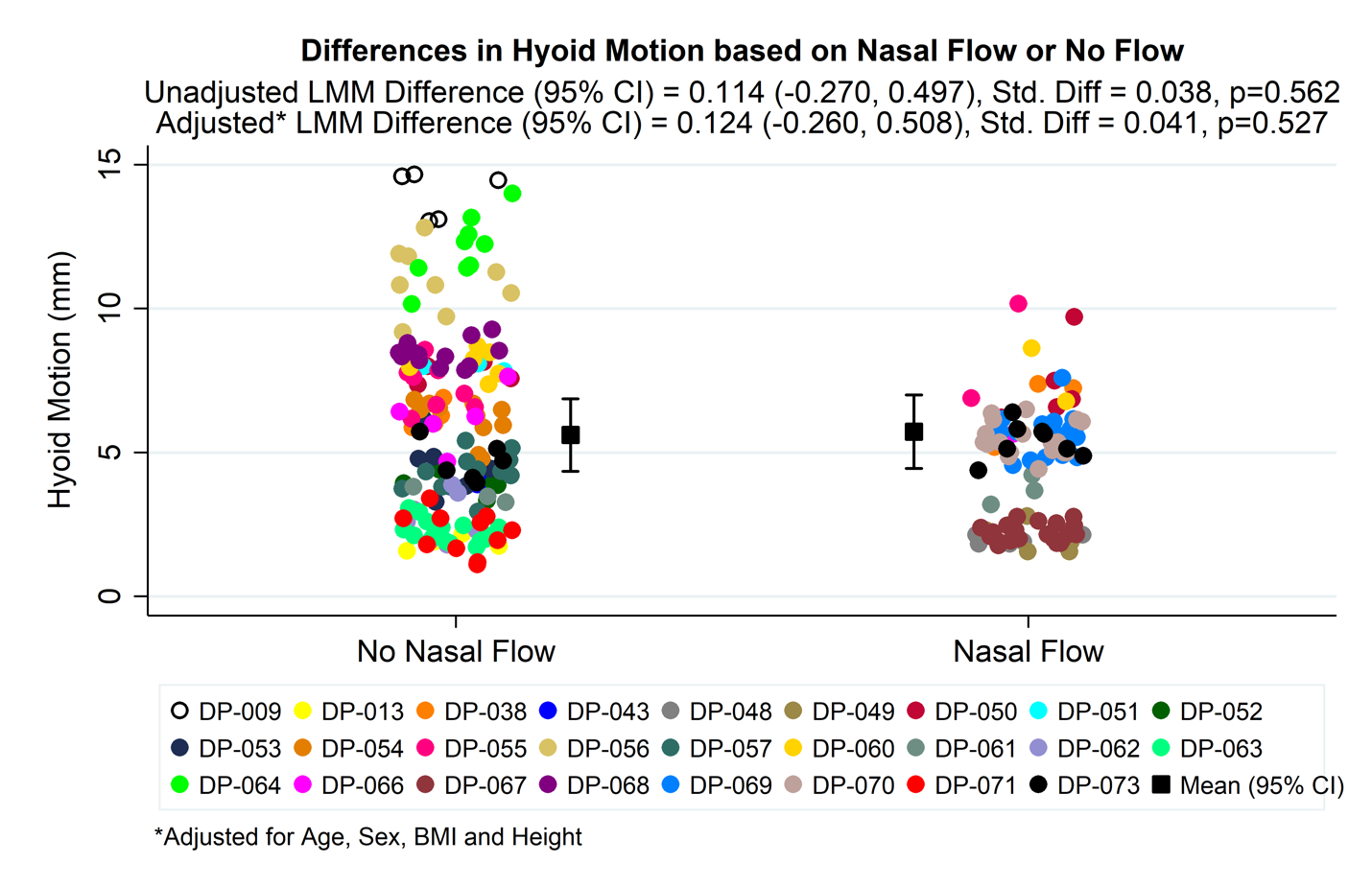
**

Figure S6: Association between Nasal Airflow and Hyoid Motion. X axis shows categorization of airflow during obstructive breathing (absence/presence). Y axis shows individual points of measured breaths of hyoid motion (mm).

**S7:** Craniofacial Measurements and Caudal Hyoid Displacement Amplitude

|  | **Adjusted for Age, Sex, BMI, Height** | | |  |
| --- | --- | --- | --- | --- |
| **Variable** | **Beta** | **Std. Beta** | **p** | |
| *Head Anteriorization (C4-sella-nasion angle) (˚)* | 0.19 (-0.07, 0.45) | 0.32 (-0.12, 0.77) | 0.14 | |
| *Head Extension (FMP-atlas plane angle) (˚)* | 0.04 (-0.29, 0.37) | 0.06 (-0.42, 0.54) | 0.81 | |
| *SNA (˚)* | 0.12 (-0.32, 0.57) | 0.13 (-0.33, 0.59) | 0.57 | |
| *Nasion-A Distance (mm)* | 0.24 (-0.17, 0.64) | 0.32 (-0.22, 0.86) | 0.23 | |
| *SNB (˚)* | -0.18 (-0.75, 0.38) | -0.15 (-0.62, 0.31) | 0.51 | |
| *Nasion-B Distance (mm)* | 0.05 (-0.17, 0.27) | 0.12 (-0.38, 0.62) | 0.63 | |
| *Cranial Base Angle (˚)* | 0.12 (-0.18, 0.42) | 0.18 (-0.28, 0.64) | 0.42 | |
| *Posterior Cranial Base (mm)* | -0.20 (-0.58, 0.19) | -0.24 (-0.70, 0.22) | 0.3 | |
| *Anterior Cranial Base (mm)* | -0.02 (-0.63, 0.59) | -0.02 (-0.60, 0.56) | 0.95 | |
| *Hard Palate Length (mm)* | 0.31 (-0.11, 0.73) | 0.33 (-0.11, 0.78) | 0.14 | |
| *IPMD Interalveolar Ridge Upper Jaw (mm)* | 0.32 (-0.20, 0.84) | 0.29 (-0.18, 0.75) | 0.21 | |
| *IPM Height (mm)* | 0.40 (-0.22, 1.02) | 0.29 (-0.16, 0.74) | 0.19 | |
| *IPM Area (mm^2^)* | 0.02 (0.00, 0.04) | 0.36 (-0.09, 0.80) | 0.11 | |
| *IPMD Interalveolar Ridge Lower Jaw (mm)* | 0.01 (-0.46, 0.48) | 0.01 (-0.51, 0.53) | 0.96 | |
| *IMD Interalveolar Ridge Upper Jaw (mm)* | 0.07 (-0.32, 0.46) | 0.08 (-0.40, 0.56) | 0.72 | |
| *IM Height (mm)* | -0.25 (-0.82, 0.31) | -0.21 (-0.67, 0.25) | 0.36 | |
| *IM Area (mm^2^)* | -0.01 (-0.03, 0.01) | -0.14 (-0.60, 0.32) | 0.54 | |
| *IM Distance Lingual-Alveolar Ridge Lower Jaw (mm)* | -0.16 (-0.56, 0.25) | -0.18 (-0.66, 0.29) | 0.43 | |
| *Upper Jaw Axial Area (mm^2^)* | 0.00 (-0.01, 0.01) | -0.08 (-0.56, 0.40) | 0.72 | |

Table S7: (n=23) Adjusted for age, sex, BMI, and height. Association between various craniofacial measurements and mean hyoid displacement. FMP = Foramen Magnum Position. SNA = sella-nasion-A plane angle. SNB = sella-nasion-B plane angle. IPMD = Interpre-molar Distance. IMD = Inter-molar distance.
